# S185. DTNBP1 IS ASSOCIATED WITH THE AGE AT ONSET OF KOREAN PATIENTS WITH SCHIZOPHRENIA

**DOI:** 10.1093/schbul/sby018.972

**Published:** 2018-04-01

**Authors:** Kyuyoung Lee, Eun-jeong Joo

**Affiliations:** 1 Eulji General Hospital, Eulji University School of Medicine; 2 Eulji General Hospital

## Abstract

**Background:**

The dysbindin gene (DTNBP1) is located in chromosome 6p22.3, one of the regions of positive linkage for schizophrenia. In particular, dysbindin protein has been found to play a role in the glutatmate neural transmission in the brain. A strong genetic association between DTNBP1 and schizophrenia has been replicated through many recent studies. However, we have not replicated positive association between DTNBP1 and schizophrenia in our Korean sample. Because schizophrenia has been regarded as a disease with a quite heterogeneous origin and evolvement, it is useful to categorize patients with schizophrenia into relatively homogeneous subsets based on clinical characteristics including age at onset (AAO). We investigated the association between DTNBP1 and AAO of schizophrenia.

**Methods:**

We assessed age at first occurrence of positive psychotic symptoms of 197 patients with schizophrenia with DSM IV diagnosis, which was re-evaluated by Korean version of Diagnostic Interview for Genetic Study. Five SNPs, SNPA, P1763, P1320, P1635 and P1655 of DTNBP1 were genotyped and genetic association analyses were performed using the PLINK program.

**Results:**

In SNPA, patients with AT (N=10) showed significant earlier AAO than those with AA (N=187) (p<0.0001). The patients with heterozygote for SNP P1763 (TG, N=40) or P1320 (CT, N=41) also showed significant earlier AAO than those with homozygote (P<0.0001, P<0.0001, respectively). In addition, haplotype of all SNPs (SNPA-P1320-P1635-P1655-P1763) analysis showed significant association with AAO (p=0.000953).

**Discussion:**

In conclusion, although we were unable to support an association between DTNBP1 and schizophrenia, DTNBP1 might play a role in disease modifying. However, considering the several limitations of this study, further research involving different polymorphisms in DTNBP1 and various clinical subsets with sufficient numbers will be required to evaluate the contribution of DTNBP1 to schizophrenia.

